# Glutathione peroxidase 4 expression predicts poor overall survival in patients with resected lung adenocarcinoma

**DOI:** 10.1038/s41598-022-25019-2

**Published:** 2022-11-28

**Authors:** Chao-Yu Liu, Chen-Chi Liu, Anna Fen-Yau Li, Tien-Wei Hsu, Jiun-Han Lin, Shih-Chieh Hung, Han-Shui Hsu

**Affiliations:** 1 Faculty of Medicine, School of Medicine, National Yang Ming Chiao Tung University, Taipei, Taiwan; 2 Division of Thoracic Surgery, Department of Surgery, Far-Eastern Memorial Hospital, New Taipei City, Taiwan; 3 Division of Traumatology, Emergency Department, Taipei Veterans General Hospital, Taipei, Taiwan; 4 Division of Thoracic Surgery, Department of Surgery, Taipei Veterans General Hospital, Taipei, Taiwan; 5 Institute of Emergency and Critical Care Medicine, National Yang Ming Chiao Tung University School of Medicine, No. 201, Sec. 2, Shih-Pai Road, Beitou District, Taipei, 11217 Taiwan; 6 Department of Pathology, Taipei Veterans General Hospital, Taipei, Taiwan; 7 Institute of Biomedical Sciences, Academia Sinica, Taipei, Taiwan; 8 Integrative Stem Cell Center, Department of Orthopedics, China Medical University Hospital, Taichung, Taiwan; 9 Graduate Institute of New Drug Development, Biomedical Sciences, China Medical University, Taichung, Taiwan

**Keywords:** Lung cancer, Biomarkers

## Abstract

This study aimed to evaluate the protein expression of glutathione peroxidase 4 (GPX4) in resected non-small cell lung cancer (NSCLC). The clinical relevance and prognostic significance of GPX4 expression were analyzed. We reviewed patients with resected NSCLCs at Taipei Veterans General Hospital between September 2002 and January 2018. Available paraffin-embedded specimens were retrieved for immunohistochemistry (IHC) staining to detect GPX4 expression. The cutoff value for defining GPX4 positivity was determined according to the percentage of tumor stained in the microscopic field. The correlation between immune expression, clinicopathologic data, overall survival (OS), and disease-free survival (DFS) were analyzed. A total of 265 NSCLC specimens were retrieved for IHC staining. GPX4 expression positive was in 192 (72.5%) according to a cutoff value of 5%. GPX4 was a significant prognostic factor for OS and DFS on multivariate analysis at both 5% and 25% cutoff values. GPX4 expression was associated with poor OS and DFS, especially in lung adenocarcinoma (*p* = 0.008, and 0.027, respectively). In conclusions, IHC analysis revealed that GPX4 expression was associated with poor survival outcomes in patients with resected lung adenocarcinoma. Further research is needed to understand the role of GPX4 in tumorigenesis and the underlying mechanism responsible for survival outcomes in patients with resected lung adenocarcinoma.

## Introduction

Lung cancer remains the leading cause of cancer death^1^. Non-small cell lung cancer (NSCLC) accounts for 85% of cases of lung cancer, of which lung adenocarcinoma (LUAD) and lung squamous cell carcinoma (LUSC) are the most common subtypes. LUAD is the most common type of NSCLC and accounts for approximately 40% of lung cancers^2^. We have seen substantial progress in targeted therapy and immunotherapy for lung cancer with a more individualized approach in the past decades^3^. Despite advances in treatment options for lung cancer in the past two decades, the prognosis for patients with lung cancer is still unsatisfactory^4^. The identification of predictive and prognostic biomarkers that can help define the most appropriate treatment for NSCLC patients remains an unmet medical need.

Oxidative stress, an imbalance between reactive oxygen species (ROS) and antioxidant defense mechanisms in the cell, is involved in several physiological and pathological processes, including carcinogenesis. Glutathione peroxidases (GPXs) are an enzyme family with peroxidase activity that protects organisms from oxidative damage by reducing lipid hydroperoxides and free hydrogen peroxide^5^. The GPX family member glutathione peroxidase 4 (GPX4), the only isoenzyme that can reduce phospholipid hydroperoxide^6^, was considered one of the most important antioxidant enzymes in mammals^7^. There are few studies that suggest that the role of GPX4 in human cancer is complex and paradoxical^8–17^. It can act as a tumor suppressor^8–12^ and an oncogene^13–17^, according to published reports.

In NSCLC, recent reports suggest that human lung cancer cell growth can be inhibited by GPX4-related ferroptosis, a type of cell death characterized by lethal accumulation of lipid-based ROS^17,18^. In other words, GPX4 could help cancer cells evade death by eliminating oxidative stress, subsequently causing progression and poor prognosis. A recent report showed that high GPX4 mRNA expression correlated with poor prognosis in LUAD^19^. However, it is not clear if GPX4 can serve as a biomarker with clinical relevance to stratify high-risk patients, and predict their outcomes. In this study, we evaluated the association between protein expression of GPX4 and prognosis in patients with resected NSCLC.

## Methods

### Patients

Tumor specimens were obtained from 265 patients with NSCLC who underwent lung resection between September 2002 and January 2018 at Taipei Veterans General Hospital. There were 91 females and 174 males, with a mean age of 65 years (range 35–90). These patients were diagnosed with clinical stage I-III, not pretreated with neoadjuvant chemotherapy or radiotherapy and underwent lung resection as a cure. The clinicopathologic data, tumor grade, tumor size, lymph node involvement, and the presence of distant metastases were retrospectively collected. The survival data were obtained from the Cancer Registry Database in the Taipei Veterans General Hospital (VGH). Overall survival (OS) and disease-free survival (DFS), defined as the time from operation to death and recurrence, respectively, were used as surrogates of prognosis. This study was approved by the institutional review board (IRB) of the Taipei VGH, which waived the requirement for individual written patient consent (IRB approval No. 2019-11-011AC, on Nov 20, 2019). All methods were carried out in accordance with relevant guidelines and regulations.

### Immunohistochemical (IHC) staining

IHC staining was performed on 4-μm-thick sections of formalin-fixed, paraffin-embedded tissue. After deparaffinization and rehydration, all sections were treated with microwaves in 10 mmol/L citrate buffer (pH 6.0) for 10 min for antigen retrieval. Following the blocking of endogenous peroxidase activity, the specimens were incubated at 4 °C overnight with the primary antibody to GPX4 (1:200, ab125066; Abcam, Cambridge, MA, USA). After washes, sections were subsequently incubated with biotinylated secondary antibodies (K4065; Dako, USA) for 30 min at room temperature. Slides were stained using 3,3′-diaminobenzidine chromogen (DAB) solution, and counterstained with hematoxylin, followed by mounting.

**Figure 1:**
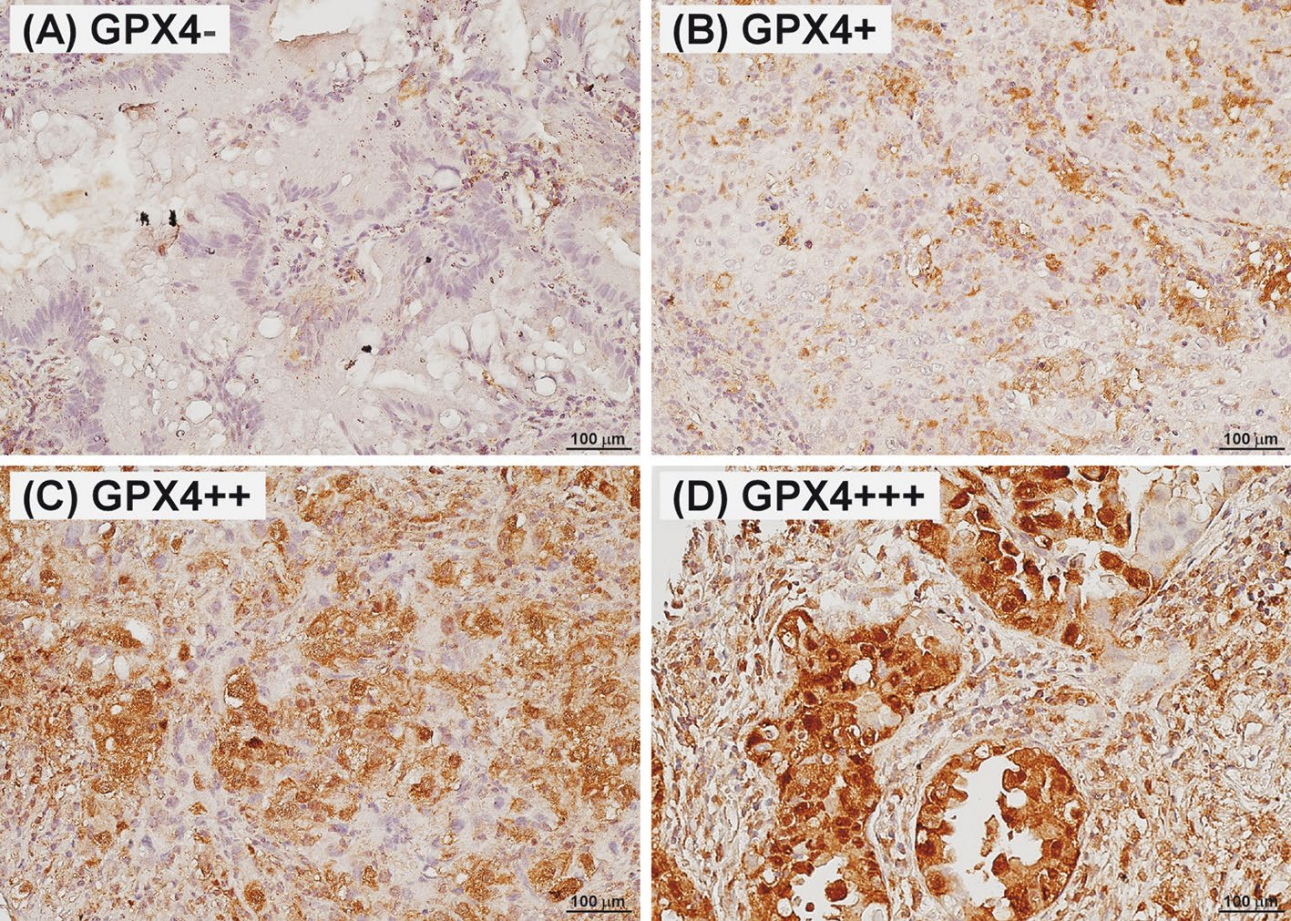
Immunohistochemical staining patterns of glutathione peroxidase 4 (GPX4) in resected non-small cell lung cancer specimens (original magnification × 400). Expression was marked and is shown in the left upper corner of each photograph as “GPX4−” if less than 5% of cells were positive (**A**), as “GPX4+” if 5–25% of cells were positive (**B**), as “GPX4++” if 25–75% of cells were positive (**C**), and as “GPX+++” if more than 75% of cells were positive (**D**) in the microscopic field.

Evaluation of IHC staining was conducted by a board-certified pathologist who was blinded to the patients’ clinical data. Since there is no consensus regarding the cutoff value of GPX4 protein expression, we determined negative if less than 5% of tumor cell were stained. On the contrary, the expression was determined positive if ≥ 5% of tumor cells were IHC-stained positive. For those with positive GPX4 expression, we categorized “GPX4^+^” if 5% to 24% of cells were positive, “GPX4^++^” if 25% to 75% of cells were positive, and “GPX4^+++^” if more than 75% of cells were positive after IHC staining. The IHC staining patterns are shown in Fig. 1.

### Statistical analysis

The correlations between IHC staining results and clinicopathological variables were analyzed by Pearson’s χ^2^ test. Survival curves were estimated by the Kaplan–Meier method, and groups were compared for outcome by the log rank test. Univariate and multivariate analysis were performed with the Cox regression model. Factors in univariate analysis with a *p-*value < 0.05 would be included for multivariate analysis with Enter Method. A *p*-value < 0.05 was significant. All calculations were performed with SPSS version 22 (IBM Corp., Armonk, NY, USA).

## Results

**Figure 2:**
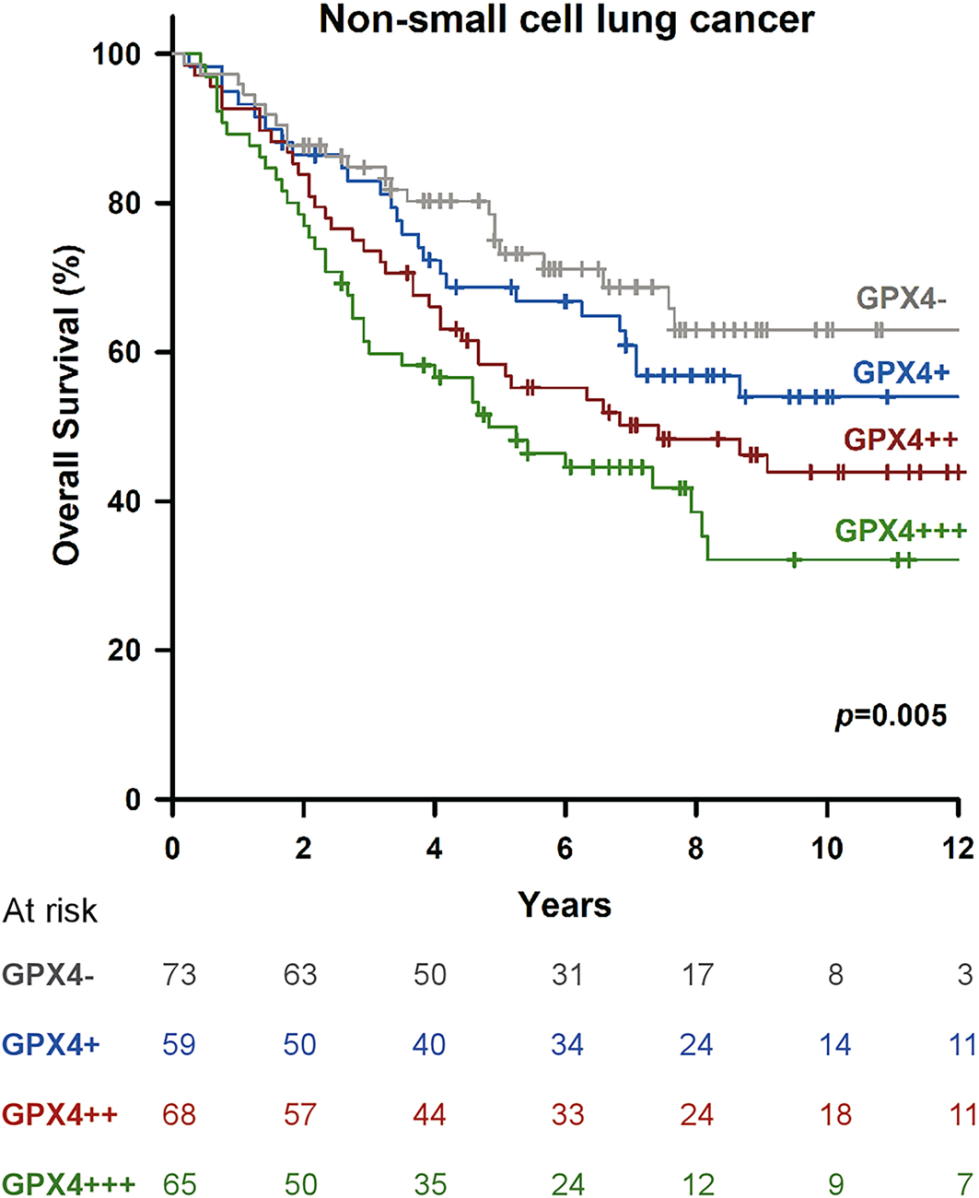
Different levels of glutathione peroxidase 4 expression, stratified by immunohistochemical stain, were significantly associated with 5-year overall survival (*p* = 0.005).

**Figure 3:**
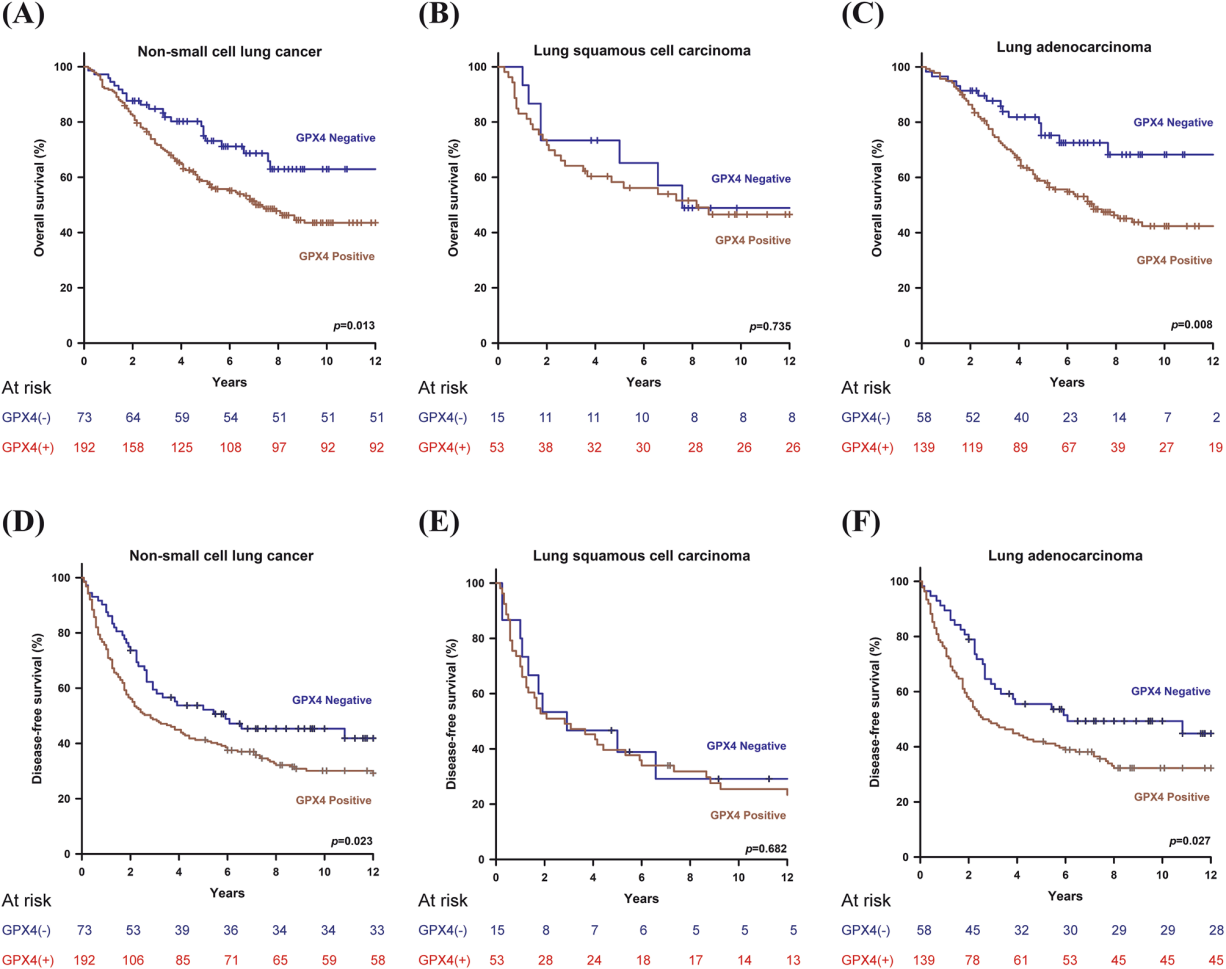
Glutathione peroxidase 4 (GPX4) expression with a 5% cutoff value was significantly associated with OS and DFS in patients with resected non-small cell lung cancer (**A** and **D**, log rank *p* = 0.013 and 0.023, respectively). In patients with resected lung squamous cell carcinoma, survival analysis showed that with or without GPX4 expression they had similar survival outcomes (**B** and **E**, log rank *p* = 0.735 and 0.682, respectively). However, in patients with resected lung adenocarcinoma, GPX4 expression had a significantly worse OS and DFS than those with negative GPX4 expression (**C** and **F**, log rank *p* = 0.008 and 0.027, respectively).

In the cohort of 265 patients with a mean age of 65.46 ± 10.94 years (range 35–90), the overall 5-year survival was 62.6%, with a median follow-up of 65 months. Demographic data and survival analysis grouped by clinicopathological characteristics are presented in Table 1. Two-third of patients were male (65.7%). According to patients’ clinical condition, pneumonectomy, bilobectomy, lobectomy, and sublobar resections were performed in 6 (2.3%), 8 (3.0%), 211 (79.6%), 40 (15.1%) patients, respectively, at operating surgeons’ discretion. After surgery, 145 (54.7%), 36 (13.6%), and 73 (27.5%) patients were in their pathological stage I, II, and III, respectively. However, 11 (4.2%) patients were upstaged to stage IV because of intraoperatively-found pleural seedings (M1) which remained resectable. Among then, 5 lobectomies and 6 sublobar resections were still performed as curative intent. The 265-formalin-fixed, paraffin-embedded surgical specimens were immunohistochemically stained to determine the GPX4 expression level. The pathologic tumor stage was determined according to the 7th edition of the American Joint Committee on Cancer staging system. For patients with pathological stage I_A_ and I_B_ with tumor < 3 cm, no further treatment was given. For patients with pathological stage I_B_ with tumor ≥ 3 cm, daily oral chemotherapy with Ufur (Tegafur 100 mg + Uracil 224 mg) would be given as adjuvant therapy for 2 years. For patients with stage II and above, adjuvant chemotherapy ± radiotherapies were given. Tyrosine-kinase inhibitors (TKIs) were selectively given to patients with advanced diseases or those having disease recurrence after primary treatments. In total, 104 (39.8%) patients had adjuvant therapies after pulmonary resections, 136 (51.3%) patients had disease recurrence during follow-ups, and 112 (42.3%) patients had post-recurrence therapies. Of the 265 resected lung specimens, there were 197 adenocarcinomas versus 68 squamous cell carcinomas. In all, 29 (10.9%), 122 (46.0%), and 114 (43.0%) of the 265 tumors were respectively well, moderately, and poorly differentiated. Protein expression of GPX4 ≥ 5% of tumor cell detected by IHC staining was defined “positive” and observed in 192 (72.5%) specimens. In total, we have 65 GPX4^+++^, 68 GPX4^++^, 59 GPX4^+^, and 73 GPX4^-^ according the definition described earlier. We have demonstrated GPX4 expression was significantly linked to 5-year survival (*p* = 0.005) (Fig. 2). There was no significant correlation between GPX4 expression and clinicopathologic factors in resected lung specimens at a cutoff value of 5%. However, we observed male patients (*p* = 0.001), smoker (*p* < 0.001), and LUSC (*p* < 0.001) were associated with positive GPX4 expression as we defined GPX4 expression at a cutoff value of 25% (Table 2). For clinicopathological factors, older age (*p* = 0.001), male patients (*p* < 0.001), smoker (*p* = 0.002), sublobar resections (*p* = 0.002), Tumor grade (*p* = 0.012), “T” (*p* = 0.005), “N” (*p* < 0.001), “M” (*p* = 0.006), and GPX4 (*p* = 0.017 and 0.002, at 5% and 25% cutoff, respectively) were associated with poor OS in univariate analysis. In multivariate analyses, only age, surgical resection, tumor grade, “T”, “N”, and GPX4 expression remained significant prognostic factors for OS with GPX4 cutoff value at both 5% and 25% (Table 3). As our definition of GPX4 expression, patients with GPX positive had worse OS and DFS than those with GPX negative (Fig. 3A,D, log rank *p* = 0.013 and 0.023, respectively). We further sub-grouped our cohort into LUSC and LUAD. In patients with resected LUSC, survival analysis showed that patients with positive or negative GPX4 expression had similar survival and recurrence outcomes (Fig. 3B,E, log rank *p* = 0.735 and 0.682, respectively). However, in patients with resected LUAD, patients with positive GPX4 expression had a significantly worse OS and DFS than those with negative GPX4 expression (Fig. 3C,F, log rank *p* = 0.008 and 0.027, respectively).

**Table 1:** Patient characteristics of 265 resected NSCLCs.

Variables	Number	(%)
Age (mean ± SD)	65.46 ± 10.944	
**Smoking status**		
Never-smoker	128	48.3
Smoker	137	51.7
**Sex**		
Male	174	65.7
Female	91	34.3
**Surgical resection**		
Sublobar	40	15.1
Lobar(s) (≥ 1 lobes)	225	84.9
**Pathology**		
LUAD	197	74.3
LUSC	68	25.7
**Tumor grade**		
G1	29	10.9
G2	122	46.0
G3	114	43.0
**T**		
T1	42	15.8
T2	188	70.9
T3	18	6.8
T4	17	6.4
**N**		
N0	163	61.5
N1	33	12.5
N2	69	26.0
**M**		
M0	254	95.8
M1	11	4.2
**pStage**		
Stage I	145	54.7
Stage II	36	13.6
Stage III	73	27.5
Stage IV	11	4.2
**Adjuvant therapy**		
Yes	104	39.8
No	161	60.8
**Recurrence**		
Yes	136	51.3
No	129	48.7
**Post-recurrence therapy**		
Yes	112	42.3
No	153	57.7
**GPX4**		
GPX4 (−)	73	27.5
GPX4 (+)	59	22.3
GPX4 (++)	68	25.7
GPX4 (+++)	65	24.5

*SD* standard deviation, *T* tumor, *N* nodes, *M* metastases, *LUAD* lung adenocarcinoma, *LUSC* lung squamous cell carcinoma, *G1* well differentiated, *G2* moderately differentiated, *G3* poorly differentiated, *pStage* pathological stage, *GPX4* glutathione peroxidase 4, *GPX4* (−) less than 5% were positive for immunohistochemical (IHC) stain, *GPX4* (+) IHC stain showed 5–25% of cells were positive, *GPX4* (++) IHC stain showed 25–75% of cells were positive, *GPX4* (+++) IHC stain showed more than 75% of cells were positive.

**Table 2:** Association between GPX4 expression and clinicopathological factors in all patients.

Factors	n	5% Cutoff			25% Cutoff		
		GPX4, n (%)			GPX4, n (%)		
		Negative	Positive	*p*	Negative	Positive	*p*
**Age**				0.203			0.206
< 70	164	50 (68.5)	114 (59.4)		87 (65.9)	77 (57.9)	
≥ 70	101	23 (31.5)	78 (40.6)		45 (34.1)	56 (42.1)	
**Sex**				0.469			0.001
Female	91	28 (38.4)	63 (32.8)		58 (43.9)	33 (24.8)	
Male	174	45 (61.6)	129 (67.2)		74 (56.1)	100 (75.2)	
**Smoking**				0.784			< 0.001
Never	128	34 (46.6)	94 (49.0)		79 (59.8)	49 (36.8)	
Smoker	137	39 (53.4)	98 (51.0)		53 (40.2)	84 (63.2)	
**Pathology**				0.273			< 0.001
LUAD	197	58 (79.5)	139 (72.4)		113 (85.6)	84 (63.2)	
LUSC	68	53 (27.6)	15 (20.5)		19 (14.4)	49 (36.8)	
**Tumor grade**				0.383			0.08
G1	29	10 (13.7)	19 (9.9)		19 (14.4)	10 (7.5)	
G2/G3	236	63 (86.3)	173 (90.1)		113 (85.6)	123 (92.5)	
**T**				0.550			0.370
T1/T2	230	62 (84.9)	168 (87.5)		112 (84.8)	118 (88.7)	
T3/T4	35	11 (15.1)	24 (12.5)		20 (15.2)	15 (11.3)	
**N**				0.262			0.900
N0	163	49 (67.1)	114 (59.4)		82 (62.1)	81 (60.9)	
N1–3	102	24 (32.9)	78 (40.6)		50 (37.9)	52 (39.1)	
**M**				0.733			0.540
M0	254	71 (97.3)	183 (95.3)		128 (97.0)	126 (94.7)	
M1	11	2 (2.7)	9 (4.7)		4 (3.0)	7 (5.3)	
**pStage**				0.558			0.895
I/II	181	52 (71.2)	129 (67.2)		91 (68.9)	90 (67.7)	
III/IV	84	21 (28.8)	63 (32.8)		41 (31.1)	43 (32.3)	

*GPX4* glutathione peroxidase 4, *n* number, *T* tumor, *N* nodes, *M* metastases, *LUAD* lung adenocarcinoma, *LUSC* lung squamous cell carcinoma, *G1* well differentiated, *G2* moderately differentiated, *G3* poorly differentiated, *pStage* pathological stage.

**Table 3:** Univariate and multivariate analysis of overall survival in resected NSCLC patients with the GPX4 protein expression at 5% and 25% cutoff values.

Factors	Univariate		5% Cutoff		25% Cutoff	
			Multivariate		Multivariate	
	HR (95% CI)	*p*	HR (95% CI)	*p*	HR (95% CI)	*p*
Age	1.025 (1.010–1.041)	0.001	1.025 (1.009–1.041)	0.002	1.027 (1.011–1.043)	0.001
**Sex**		< 0.001		0.207		0.175
Female	1		1		1	
Male	1.916 (1.345–2.730)		1.338 (0.852–2.101)		1.365 (0.871–2.141)	
**Smoking**		0.002		0.153		0.521
Never	1		1		1	
Smoker	1.618 (1.185–2.208)		1.342 (0.897–2.009)		1.145 (0.757–1.734)	
**Surgical resection**		0.002		0.010		0.015
Lobar(s) (≥ 1 lobes)	1		1		1	
Sublobar	1.873 (1.253–2.798)		1.812 (1.153–2.848)		1.745 (1.116–2.728)	
**Pathology**		0.063				
LUAD	1					
LUSC	1.368 (0.983–1.904)					
**Tumor grade**		0.012		0.024		0.047
G1	1		1		1	
G2/G3	2.067 (1.169–3.653)		1.937 (1.091–3.439)		1.791 (1.007–3.186)	
**T status**		0.005		0.060		0.036
T1/T2	1		1		1	
T3/T4	1.816 (1.200–2.748)		1.499 (0.982–2.288)		1.578 (1.031–2.415)	
**N status**		< 0.001		< 0.001		< 0.001
N0	1		1		1	
N1/N2	2.628 (1.927–3.584)		2.799 (2.024–3.872)		2.819 (2.039–3.897)	
**M status**		0.006		0.195		0.294
M0	1		1		1	
M1	2.461 (1.290–4.694)		1.595 (0.788–3.228)		1.464 (0.718–2.985)	
**Adjuvant therapy**		0.151				
No	1					
Yes	1.256 (0.920–1.714)					
**GPX4 (5% cutoff)**		0.017		0.016		
Negative	1		1			
Positive	1.614 (1.089–2.392)		1.648 (1.099–2.472)			
**GPX4 (25% cutoff)**		0.002				0.012
Negative	1				1	
Positive	1.638 (1.199–2.238)				1.534 (1.099–2.143)	

*NSCLC* non-small cell lung cancer, *HR* hazard ratio, *CI* confidence interval, *GPX4* glutathione peroxidase-4, *T* tumor, *N* nodes, *M* metastases.

## Discussion

GPXs have been known to catalyze the reduction of H_2_O_2_ or organic hydroperoxide to water or the corresponding alcohols using glutathione (GSH) as a reductant. GPXs include eight members in different body tissues with different functions. In cancer, GPX1 and GPX3 are tumor suppressors, while GPX2 may have dual roles in tumorigenesis^20^. GPX4, a selenoprotein, is the only known antioxidant enzyme that can directly reduce peroxidized phospholipid and cholesterol in membranes, and use a wide range of reducing cofactors in addition to GSH^21^. Almost all normal mammalian cells show GPX4 activity, particularly abundant in testis, adipose tissue, and retina^22^. In tumors, the role of GPX4 has been ambiguous and paradoxical. GPX4 expression was increased in renal cell carcinoma^14^, hepatocellular carcinoma^15^, and colon cancer^23^ but decreased in pancreatic cancer^9^, breast cancer^10^, renal cell carcinoma^24^, gastric cancer^25^, hepatocellular carcinoma^11^, and human astrocytoma^12^. This finding means that in different types of cancer, even in the same type, GPX4 can take on different roles regulating tumorigenesis^17^. There are fewer reports on the role of GPX5, -6, -7, and -8 in tumorigenesis^19,20^.

The association between GPX4 and cancer OS has been investigated. GPX4 expression was found negatively associated with OS in cholangiocarcinoma, colon cancer, and LUSC patients. However, in patients with hepatocellular carcinoma, skin melanoma, testicular germ cell tumor, uterine carcinosarcoma, there was no significance between GPX4 and OS^17^. In a study investigating the association between GPX4 and OS in patients with diffuse large B cell lymphoma (DLBCL), the GPX4-positive group had a significantly poorer prognosis in OS. However, there was no significant difference in mRNA levels between the GPX4-positive group and GPX4-negative group, suggesting that post-transcriptional regulation may be involved in GPX4 protein expression in DLBCL^13^. Liu et al., using an online database, reported that high GPX4 mRNA expression correlated with OS in LUAD patients^19^. To the best of our knowledge, no study has yet assessed the relationship between GPX4 protein expression and OS in patients with resected NSCLC. In our study, 192 (72.5%) patients were positive for GPX4 protein expression, and these patients showed a significantly poor prognosis in OS and DFS. Moreover, GPX4 was an independent prognostic factor for OS and DFS. These results suggest that GPX4 expression might result in poor prognosis by a different mechanism than the existing known prognostic predictors of lung cancer, such as TNM staging.

Ferroptosis, a unique form of regulated cell death first reported by Dr. Brent R. Stockwell in 2012, describes a non-apoptotic form of iron-dependent oxidative cell death^26^. This lethal process is mediated by the accumulation of lipid peroxidation products derived from iron metabolism with subsequent depletion of plasma membrane components, including cholesterol and polyunsaturated fatty acids^27^. In the context of cancer, evidence indicates that cancer cells produce a higher level of ROS compared to normal cells, suggesting that it might be possible to eliminate cancer cells by modulating their redox status^28^. GPX4, a known negative regulator of ferroptosis, may play a crucial role in preventing cells from ferroptosis. The hypothesis of whether NSCLC, especially LUAD, is sensitive to ferroptosis remains an open question. If this hypothesis can be applied to our results, the poor prognosis of LUAD with GPX4 expression may be due to the suppression of ferroptosis in cancer cells. In other words, GPX4-expressing tumor cells are likely to acquire the ability to detoxify ROS, and be resistant to therapy. This theory was echoed by a study that showed erastin, a ferroptosis inducer, decreased NSCLC cell radioresistance by inducing GPX4-mediated ferroptosis^18^. We would need to verify this point by observing whether cell death increases under treatment with GPX4 inhibitors in future studies.

There has been no consensus regarding the cutoff values of GPX4 protein expression predicting overall survival of NSCLCs. The reason why we defined a positive GPX4 expression at a cutoff value of 5% is that it is easier to differentiate a stained specimen (≥ 5%) from a barely or non-stained one (< 5%). Technically, there is much ambiguity to differentiate among less-stained specimens (5–24%), moderately-stained specimens (25–75%), and more-stained ones (> 75%). In our study, the association between GPX4 expression and overall survival at a 25% cutoff value (*p* = 0.010) was statistically stronger than it was at a 5% cutoff value (*p* = 0.048) (Table 3). Although, it seemed reasonable to set GPX4 expression at a 25% cutoff value in our study when it comes to better differentiating survival outcomes, we were not eligible to say which cutoff was the best.

In our study, GPX4 expression was not prognostic in LUSC patients. We have not yet known the reason. However, we noted that smoker, male patients, and LUSC were associated with positive GPX4 expression when we defined GPX4 positive at a cutoff value of 25% (Table 2). Since smoking was known to be associated with ferroptosis and GPX4 expression^29^, we believed that the association between smoking and LUSC may be one of the potential reasons why no correlation could be observed between GPX4 expression and LUSC in this study.

There are still several unresolved problems and limitations in this study. First, this study was unable to elucidate the underlying mechanism causing the poor prognosis in patients with GPX4 positive NSCLC. GPX4, an antioxidant enzyme, may play an important role in carcinogenesis in the context of lung cancer. However, the exact roles of oxidative stress and antioxidant in carcinogenesis have been controversial because oxidative stress can promote or suppress tumor development in different contexts^30^. The relationships between oxidative stress and the redox system in cancer can be complex. There is no consensus on the relationship between GPX4 expression and patient survival in lung cancer. Second, ferroptosis prevented by GPX4 might be one of the mechanisms that explain the relative immortal nature of cancer cells that contributed to poor prognosis in patients with GPX4-positive NSCLC in this study. However, the causal relationship between ferroptosis and survival is not yet proven herein. Third, the prognostic role of GPX4 for LUSC was not demonstrated in this study. It can be explained by a relatively small group of LUSC patients recruited for analysis or explained by the interaction between smoking and GPX4 expression in LUSC group. Fourth, the cohort of the present study included only patients with resected NSCLCs. The results cannot be well-applied to whole NSCLC groups in which the majority was unable to have lung resection as a treatment option.

In conclusion, using IHC analysis, we found that GPX4 expression is independently associated with poor OS and DFS in NSCLC, especially in LUAD. GPX4 is a prognostic predictor and has the potential to become an anticancer drug target for NSCLC treatment. Further investigation is needed to understand the roles of GPX4 in lung cancer tumorigenesis and its underlying mechanisms responsible for survival.

## Data Availability

All data analysed during this study are included in this published article.
